# Global Risk Maps of Climate Change Impacts on the Distribution of *Acinetobacter baumannii* Using GIS

**DOI:** 10.3390/microorganisms11092174

**Published:** 2023-08-28

**Authors:** Amal Sabour

**Affiliations:** Department of Botany and Microbiology, College of Science, King Saud University, P.O. Box 2455, Riyadh 11451, Saudi Arabia; amsaboor@ksu.edu.sa

**Keywords:** biogeography, MaxEnt, bioclimatic variables, global warming

## Abstract

Impacts of climate change rank among the century’s most significant ecological and medical concerns. As a result of climatic changes, the distribution of some bacterial species will alter across time and space. Numerous bacterial infections will reorganize as a result worldwide. *Acinetobacter baumannii* Bouvet and Grimont is one of the most significant and frequently occurring bacteria identified in soil and air. The COVID-19 pandemic has changed how bacteriologists perceive this species as a new threat to human health. In order to estimate the existing and future worldwide distribution of *A. baumannii* under various climate change scenarios, about 1000 *A. baumannii* occurrence records were employed. Given its superior accuracy and dependability versus alternative modeling techniques, maximum entropy implemented in MaxEnt was selected as the modeling tool. The bioclimatic variable that contributes the most to the distribution of *A. baumannii* is the mean temperature of the coldest quarter (bio_11). The created current distribution model agreed with the species’ actual globally dispersed distribution. It is projected that *A. baumannii* will experience a severe range expansion due to the increase in temperature brought on by global warming in different regions of its range. According to the risk maps created for 2050 and 2070 using two alternative RCPs, there are various regions that will be under risk of this bacterium as a result of rising temperature. Future data science and GIS evaluation of the current results are necessary, especially on a local level.

## 1. Introduction

*Acinetobacter baumannii* is a bacterial species that is prevalent in soil and water. It is also an opportunistic pathogen that can cause deadly infections in people, particularly in hospitals [1]. *A. baumannii* is well-known for its capacity to live in a variety of environments, including surfaces and medical equipment [2]. This makes it especially difficult to control and cure in hospital settings, where it can spread easily from patient to patient. Infections produced by *A. baumannii* can range from mild to severe, including pneumonia, meningitis [3], and bloodstream infections [4]. Multiple antibiotics, including carbapenems, which are often employed as a last line of defense against bacterial infections, are frequently resisted by this bacterium [5]. Although slightly less common than healthcare-associated infections, *A. baumannii* is a ubiquitous pathogen that is also known to cause community infections that can easily cause large outbreaks [6].

*Acinetobacter baumannii* has emerged as a major global pathogen as a result of a number of causes, including its capacity to live in a variety of conditions, its potential to acquire antibiotic resistance, and the widespread use of invasive medical devices in healthcare settings [2,5]. Global travel and migration have also contributed [7] to the spread of *A. baumannii*, as the bacteria can be carried by persons who are colonized or infected [8]. The spread of the COVID-19 pandemic has also contributed to the emergence of *A. baumannii* as a global health issue [9]. *A. baumannii* secondary infections have been frequently reported in COVID-19 patients, particularly those who are hospitalized and require mechanical ventilation [10]. This could be attributed to a number of circumstances, including the use of invasive medical devices, prolonged hospitalization, and a weakened immune function.

Furthermore, there are worries that the increased use of antibiotics [9] to treat COVID-19 patients may lead to the development of antibiotic-resistant infections, such as those caused by *A. baumannii*, because the bacterium is notorious for developing resistance to numerous drugs [11], including but not limited to those in many different antibiotic families such as beta-lactams (penicillin, cephalosporin, carbapenems, monobactams, and beta-lactamase inhibitors), aminoglycosides, tetracyclines, fluoroquinolones, macrolides, lincosamides, strepgramin antibiotics, polymyxins, amphenicols, oxazolidinones, rifamycins, fosfomycin, glycopeptide, and lipopeptide antibiotics, possibly among many others [12]. Due to its very large spectrum of broad multidrug resistance, it comes as no surprise that the treatment of *A. baumannii* infection is currently one of the most difficult among nosocomial infections [13].

Given the nature of nosocomial infections and the vulnerability of the patients suffering from co-infection or an otherwise weakened immunity, one small study found that the mortality rate of patients who acquired an *A. baumannii* bloodstream infection while enrolled in hospital was as high as 29%, with the highest risk factor shown to be neutropenia after multivariate analysis [14]. Another systematic review found that the infection mortality rate of *A. baumannii* was as high as 43% in patients admitted to the intensive care unit [15].

In addition to the aforementioned challenges, some small studies have demonstrated that there may be some seasonal variation in the incidence of Acinetobacter infections, with some even going as far as to suggest that climate change may directly influence its epidemiology [16]. Climate change in general is already one of most serious current global concerns [17], which is mostly driven by anthropogenic activity, mainly the burning of fossil fuels [18], changes in land usage [19], and solid waste landfills [20]. Some of the impacts of climate change are observable in the form of changes in precipitation patterns [21], an increased likelihood of extreme weather events [22], and subsequently, an increased incidence of natural disasters [23].

Intuitively, the impacts of climate change on biodiversity are undeniable. These impacts include posing a risk of extinction for organisms that are unable to adapt [24], while those that can may do so by altering behavior, morphology, and/or physiology [25], which may lead to range shifting [26], which itself poses a risk for a non-indigenous species becoming invasive [27]. For all these reasons, climate change can ultimately lead to ecological imbalances that may have notorious ecological and economic consequences [28].

Fortunately, with the rise in modern computing came the development of the geographic information system (GIS) [29]. A GIS is defined as any computer system that can capture, store, retrieve, analyze, and/or visualize geospatial data [30]. This has proven revolutionary to remote sensing technology, birthing what is known as integrated GIS, which aims to ease the integration between GIS technologies with remote sensing technologies [31].

Combining high-throughput GIS analysis techniques with high-throughput remote sensing data generated by modern satellites has enabled us to study many different characteristics of the Earth remotely, thus eliminating the need for field-based and invasive experiments, allowing us to infer the biophysical properties of species’ habitats [32], monitoring land cover, its changes, and their effects on biological systems [33] and, most importantly, within meteorology and climate studies [29]. Remote sensing can also be used to directly track individuals’ movement via sensors in a technique known as active remote sensing [34] to study the migration, distribution, and behavior of a species.

Geographic information systems, remote sensing technology, the understanding of the climate, and the rise of data science powered by widely available data and machine learning algorithms have enabled the creation and study of mathematical and computational climate models [35,36]. This led to the development of a tool known as species distribution modeling (SDM). Species distribution modeling algorithms correlate known-occurrence records of a species with environmental data to predict said species’ potential distribution across other geographical areas [37]. Species distribution models have myriad applications, including but not limited to biogeography, conservation biology, climate change research [37], and invasive species management [38].

This study aims to shed light on the vague relationship between the climate and *A. baumannii* epidemiology [16], as well as predict for the first time for bacterial species the potential global distribution under different climate change scenarios in 2050 and 2070, by employing species distribution modeling on historic and future projected climate data.

## 2. Materials and Methods

### 2.1. Data Collection

#### 2.1.1. Species Occurrence Records

Species occurrence records for *Acinetobacter baumannii* were downloaded in the Darwin Core (DwC) format from the Global Biodiversity Information Facility (GBIF) [39]. Data obtained from GBIF include data recorded in the literature, as well as data submitted by users who volunteer to contribute to public databases [40].

**Figure 1:**
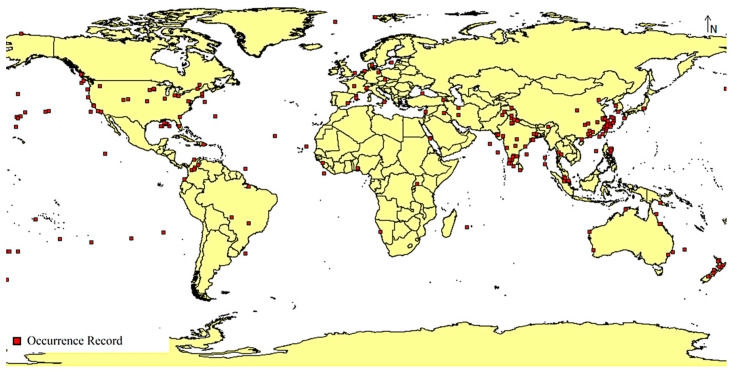
World map showing occurrence records of *A. baumannii*.

The occurrence records were filtered out to only include those with longitude and latitude coordinates recorded, and then further filtered out to remove suspicious unverified occurrence records. The longitude and latitude coordinates were then saved in the CSV file format for compatibility with the MaxEnt application that was used for modeling. A total of 1044 final occurrence records were used in this study (Figure 1).

#### 2.1.2. Climate Data

Climate data represented in 19 bioclimatic variables were downloaded from WorldClim version 1.4 using a spatial resolution of 2.5 min, which corresponds to approximately 5 km at the equator (Table S1) [41]. These data were downloaded for the current climate scenario (historical mean of values from 1960 to 1990) as well as future projected scenarios in the years 2050 (projected mean of values from 2041 to 2060) and 2070 (projected mean of values from 2061 to 2080).

For future projected climate data, the general circulation model used was the MRI-CGCM3, developed by the Japanese Meteorological Research Institute (MRI) [42]. The representative concentration pathways used were RCP 2.6 and RCP 8.5 to forecast potential best- and worst-case scenarios, respectively [43].

All the data were finally converted into ASCII format using ArcMap version 10.3 to enable it to be used with MaxEnt.

### 2.2. Model Construction

Species distribution models were constructed using the maximum entropy algorithm as implemented in the MaxEnt application [44] using the species occurrence data and climate data obtained. Maximum entropy as an algorithm was chosen because of its ability to construct statistically accurate species distribution models using only species’ presence data without needing species’ absence data, which are not available for most species [45], as well as its superior accuracy and dependability as compared to other species distribution modeling algorithms.

First, a preliminary model was constructed using all 19 historic bioclimatic variables to determine the variables that are the most powerful contributors to the model or the most powerful predictors. The use of bioclimatic factors 8, 9, 18, and 19 was avoided during variable selection due to known spatial artifacts and discontinuities [46], which may adversely impact the model quality. The factors were selected through an interpretation of the response curves as well as the jack-knife test. The response curves show the predicted probability of suitability for *A. baumannii* in given environmental variable ranges, while the jack-knife test is a method of assessing the significance or importance of the used environmental features, which depends on constructing models using all variables, omitting one in turn. It then estimates the ability of the model to make predictions, omitting the excluded variable, and the larger the differences between the models, the more important the predictor is deemed [47].

Next, a model for the potential current distribution of *A. baumannii* was constructed using the same species occurrence records but with only the selected bioclimatic variables, deemed the most powerful environmental predictors.

Then, for the potential future distribution, a model was constructed using climate data for each of the four used scenarios (RCP 2.6 and 8.5 for each of 2050 and 2070) using the same selected bioclimatic variables. The future climate data for each run were loaded into MaxEnt’s projection layers for suitability in the current climate scenario to be estimated, but then projected against the future climate data.

For all the aforementioned models, the settings used on the MaxEnt application were as follows: 10 replicate runs, response curves and jack-knife testing enabled, cloglog output format, 25% random testing data, and 75% random training data. Accounting for the random differences between the replicate models, the median was used as the final result for each run. The output from each model is an ASCII raster world map consisting of values ranging from 0 to 1, where each value is interpreted as the probability of a given point being environmentally suitable for the species.

### 2.3. Quality Assessment

The model quality was assessed according to the area under the receiver operating characteristics curve (AUC) [45] and the true skill statistic (TSS) [48]. The mean AUC across the models is automatically exported by MaxEnt itself and is a threshold-independent metric that ranges from zero to one, where 0.9–1 signifies excellent quality, 0.8–0.9 signifies good, 0.7–0.8 signifies satisfactory, 0.6–0.7 signifies poor, and less than 0.6 signifies that the model failed [49].

The true skill statistic, on the other hand, is a threshold-dependent metric calculated as the sum of the true positive rate and true negative rate minus 1, where the value ranges from −1 to 1. A TSS value of positive one indicates a hypothetical model that perfectly distinguishes between positives and negatives, while zero or negative values indicate a model whose performance is no better than random [48]. The threshold used to classify the models into presence/absence points was 0.6, representing a 60% probability of environmental suitability. Points with probabilities greater than or equal to 0.6 were used to select presence points, and else for absence points.

### 2.4. Visualization and Exporting Maps

The generated models were each individually visualized on a world map and exported using ESRI ArcMap 10.3 [50]. They were classified into different categories using Jenks natural breaks optimization and then color-coded to each visual interpretation.

The maps were then classified into a presence–absence map using the same threshold used to calculate the TSS as previously mentioned, where zeroes represent absence points and ones represent presence points. To ease the interpretation of the future projected distribution maps, the maps were calibrated by subtracting the present–absent values of the current distribution maps from those of the future distribution maps [51]. This yielded a final raster map where positive values signify range gain, zeroes signify unaffected range, and negative values signify range loss. The classified maps were then color-coded and exported as well.

## 3. Results

### 3.1. Model Quality

Quality assessment according to the AUC value revealed that the model was of very good quality [49], with a mean AUC value of 0.869 across the replicate runs.

The TSS value was computed at 0.745, which is significantly better than the default threshold value of 0.5, revealing that the models were of satisfactory quality.

### 3.2. Significant Environmental Variables

The preliminary MaxEnt runs showed that the most significant environmental variables deemed the most powerful predictors or contributors to the model were bio_1 (annual mean temperature), bio_6 (minimum temperature of the coldest month), bio_11 (mean temperature of the coldest quarter), and bio_12 (annual precipitation). The environmental enveloped test was performed for bio_1 and bio_12 for a better understanding of the ecological requirements of this bacteria (Figure S1).

The response curves strongly suggest that *A. baumannii* has a preference for warm and wet climates.

**Figure 2:**
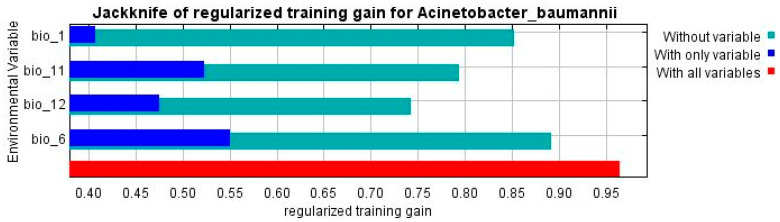
Jack-knife test of *A. baumannii* showing the most significant bioclimatic variable that affects its distribution.

**Figure 3:**
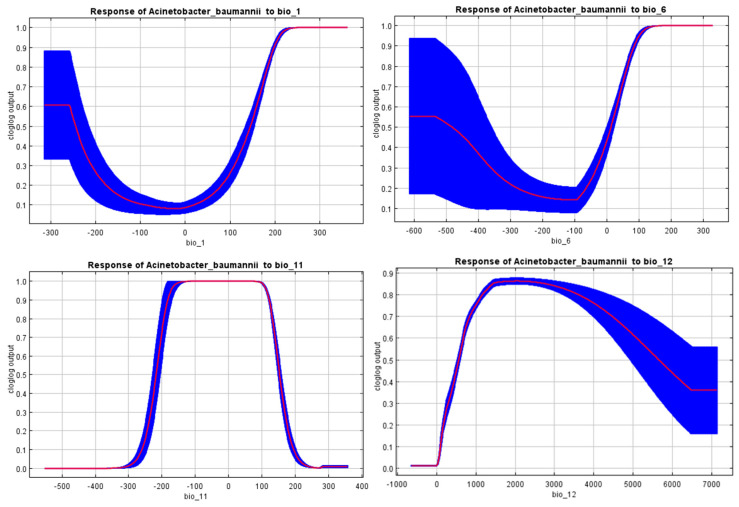
Response of *A. baumannii* to the most powerful predictors.

An analysis of the jack-knife test shown below in Figure 2, which is also used to assess variable importance or significance, revealed that the distribution of *A. baumannii* is most affected by the mean temperature of the coldest quarter (bio_11) and the minimum temperature of the coldest month (bio_6), which, when combined with the response curves shown in Figure 3, also shows a tendency to prefer warmer temperatures.

Consistent with the previous results, the MaxEnt runs also showed that the following factors contributed the most to the distribution of *A. baumannii*. Note that the permutation importance is preferred over the percent contribution when using environmental variables that are correlated with each other, such as the mean temperature of the coldest quarter (bio_11) and the minimum temperature of the coldest month (bio_6), because in such cases, the percent contribution refers mostly to the amount of information present in said variable that is not present in any other variable used [52] (Table 1).

### 3.3. Potential Current Distribution

**Figure 4:**
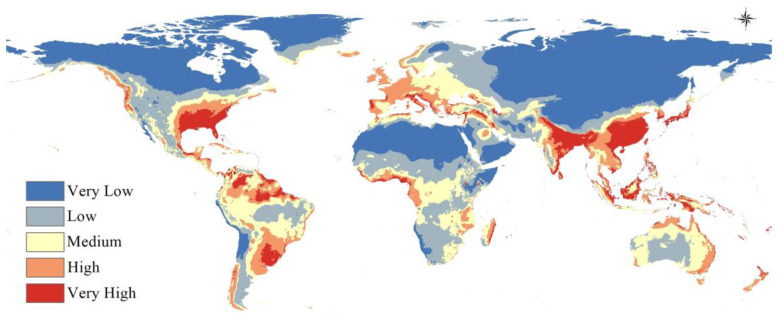
Global distribution of *A. baumannii* in the current climate scenario.

A visualization of the models constructed on historic climate data as an estimation of the current climate scenario revealed that *A. baumannii* has a significant and wide potential global distribution that can potentially include every biogeographic realm, as shown in the following figure. Warmer colors indicate a higher probability of suitability for *A. baumannii* and vice versa for colder colors (Figure 4).

**Figure 5:**
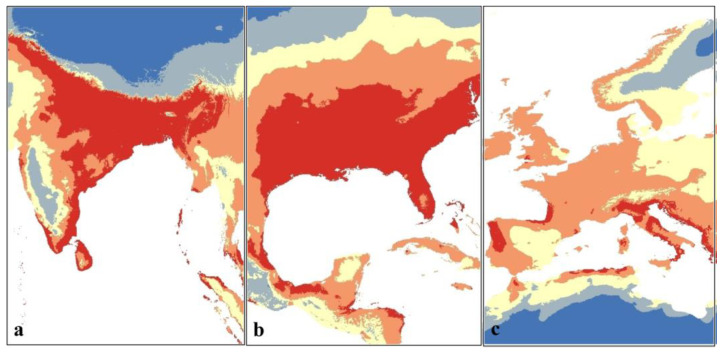
Potential current distribution of *A. baumannii* in (**a**) South Asia, (**b**) southeastern U.S., and (**c**) Europe.

In line with the response curves indicating that *A. baumannii* appears to prefer warm and moist climates, the map shows that the largest areas with the highest probability of suitability are predominantly concentrated in south Europe, South Asia, and the south-east of the United States (Figure 5). Significant potential ranges suitable for *A. baumannii* are also observed in most of South America and Australia.

### 3.4. Potential Future Distribution

**Figure 6:**
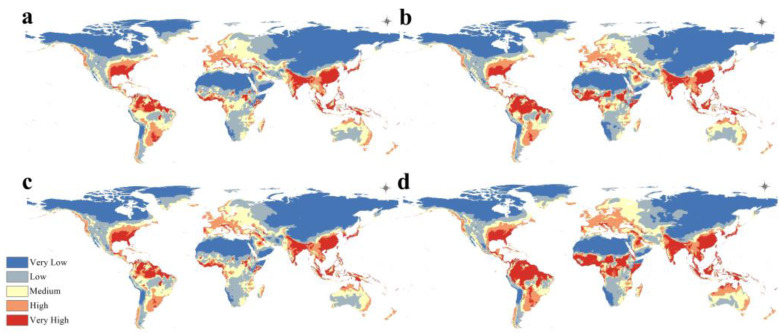
Projected global distribution of *A. baumannii* in 2050 under (**a**) RCP 2.6 and (**b**) RCP 8 and in 2070, (**c**) RCP 2.6 and (**d**) RCP 8.

The MaxEnt models built on projected climate data in 2050 and 2070 indicated that *A. baumannii* is projected to still enjoy a considerably wide range in the future in different climate change scenarios (Figure 6).

For both RCP 2.6 and RCP 8.5, there appears to be a significantly higher range suitability for *A. baumannii* when contrasted with the potential current distribution shown in Figure 3. To clarify the exact changes in range predicted to be undergone by *A. baumannii* in response to climate change, the following calibration maps will be used for result interpretation instead of the previously shown maps.

**Figure 7:**
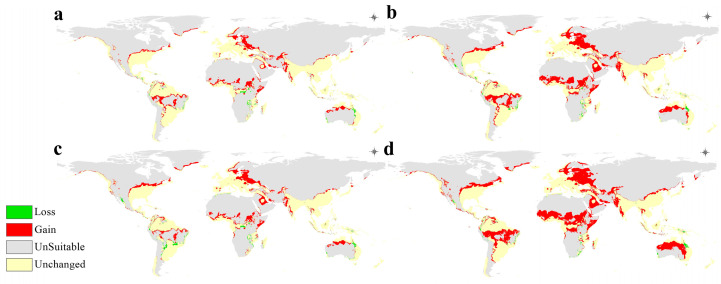
Calibration maps of the projected *A. baumannii* global distribution in (**a**) 2050 RCP 2.6, (**b**) 2050 RCP 8.5, (**c**) 2070 RCP 2.6, and (**d**) 2070 RCP 8.5.

The calibration maps (Figure 7) mostly show an alarmingly high range of expansion in all climate change scenarios used in this study. Specifically, with rising global mean temperatures and the expected associated changes in precipitation patterns, the range of *A. baumannii* is projected to expand to include new ranges in Northern and Central Europe, Sub-Saharan Africa, most of South America, Australia, and the Arabian Peninsula. This range expansion is also clearly projected to become increasingly more extreme in the more severe climate change scenarios with rising global temperatures, as can clearly be seen when comparing Figure 7d (2070 in the worst-case scenario) with Figure 7a (2050 in the best-case scenario).

The models also predicted negligible range loss, which is expected to be a form of range shifting into areas with warmer and wetter climates, as *A. baumannii* appears to prefer.

## 4. Discussion

Nosocomial infections (NIs), also known as hospital-associated or healthcare-associated infections (HAIs) are a serious threat to healthcare worldwide, affecting as many as 1 in every 10 patients admitted to hospital [53]. Among the organisms that cause nosocomial infections are *Acinetobacter* spp., with one five-year review even finding that *Acinetobacter* spp. was indeed the most common among them [54]. This has notorious consequences as *Acinetobacter* spp., especially *A. baumannii*, are capable of quickly developing multidrug resistance due to their mobile genetic elements, such as insertion sequences and transposable elements [55]. Pathogenic *Acinetobacter* spp. are also known to spread through communities and cause community-acquired infections, especially after war or natural disasters [56].

Like all other living organisms, *A. baumannii* is subject to the possible ecological impacts of climate change, that may include adaptation to the new conditions [25], which may lead to range shifting [26]. A 2018 case study suggested that the climate plays a role in how most *A. baumannii* infections are diagnosed and reported in tropical areas, and noted that there may also be some seasonal variation to its healthcare. It also suggested that climate change may influence the infection incidence, but the claim needs to be investigated more thoroughly [16].

This study aimed to shed light on the aforementioned implied correlation between climate and *A. baumannii* infection incidence. Maximum entropy modeling implemented in MaxEnt [44] was employed to construct species distribution models for *A. baumannii* in the current climate scenario to show its possible range in the present, as well as in different future climate change scenarios to predict what the distribution and inferred infection incidence may look like in the foreseeable future. The performance of the models was judged according to their mean AUC and TSS values and were deemed to be of very good quality [49]. The models also showed that the environmental factors that affected the distribution of *A. baumannii* the most were the mean temperature of the coldest quarter (bio_11) followed closely by the minimum temperature of the coldest month (bio_6), as shown in Figure 2 and Table 1. The response curves in Figure 3 from the MaxEnt models showed that *A. baumannii* indeed appears to prefer warmer climates. Through a thorough interpretation of the response curves, the jack-knife test, and the permutation importance (Table 1), it can be safely concluded that the geospatial range of *A. baumannii* is subject to the heaviest weight of seasonal temperature, with a clear preference for warmer climates.

The map of the current potential distribution of *A. baumannii* (Figure 4 and Figure 5) shows that the regions with the highest regional suitability for *A. baumannii* are predominantly concentrated in South Asia, southeastern United States, Southern Europe, and South America. This is in line with currently reported *A. baumannii* infection cases [57,58,59]. It is also in line with the observation [16] that most diagnosed and reported *A. baumannii* infections are usually located in more tropical climates rather than cooler, continental climates, which will be further clarified in the future projected distribution maps.

In the maps representing the future projected distribution of *A. baumannii* (Figure 6 and Figure 7), there is a very clear range expansion throughout all the regions where *A. baumannii* was known to be prevalent, and new significant range developments are expected in several regions, most notably the Arabian Peninsula, North and Central Europe, and Sub-Saharan Africa. This may confirm the seasonality of *A. baumannii* infection incidence as it indeed seems to prefer warmer climates. In the regions that were predicted to undergo *A. baumannii* range expansion, it can unfortunately be assumed that its infection incidence will increase in the future, which represents a growing problem, particularly for Africa and Europe [60], where *A. baumannii* infection is already known and prevalent, especially in the Mediterranean region [61].

Regions with developing or underdeveloped countries, which may have underfunded or underdeveloped healthcare facilities, may be the most adversely impacted by this projected change. Indeed, most of Sub-Saharan Africa and Central Europe [62] will need to take the most precautionary measures against *A. baumannii* infection by controlling its spread so as to not overwhelm healthcare systems. It is of major concern that in today’s accessible world and with global travel easily available, infections such as *A. baumannii* can easily transcend state borders as well, evident by the finding of antibiotic-resistant strains of *A. baumannii* in a Kenyan hospital, and upon further microbiological and genomic analysis, they were shown to be of European and Indian lineages [63]. An outbreak of *A. baumannii*, particularly in the developing world, may prove disastrous for its citizens.

These results show that *A. baumannii* infection incidence is a growing problem and with the rise in antibiotic resistance [3], treatment has proven difficult. Because most *A. baumannii* strains are multidrug-resistant, the use of multiple antibiotics often is still not sufficient to treat infection, and for this reason, it has been suggested that new therapies and more well-controlled studies are needed to study possible treatment options, as well as placing a greater emphasis on the prevention of infection transmission [13]. In line with this vision, the World Health Organization has declared *A. baumannii* a top priority for the development of new medical countermeasures, such as a vaccine, [64] and work toward such a vaccine is indeed underway, [65] although no vaccine has been developed sufficiently to the point of human clinical trials to date [66].

This study also shows that the possible ecological effects of climate change can also be disastrous from a healthcare perspective and are not only limited to natural disasters [23] or economic consequences [28]. The changes occurring in ecosystems around the world can directly affect human health, as shown with the increased *A. baumannii* infection incidence, and this may serve as an urgent warning system to promote sustainable development, as well as raise public awareness on climate change and its consequences.

I would also like to note that in this study, species distribution models for *A. baumannii* were constructed using only bioclimatic variables, which may provide a satisfactory estimation of possible geographic range, but can still be improved through the use of more environmental variables, such as soil type, considering that multidrug-resistance *A. baumannii* is known to inhabit soils [67]. Local or regional studies conducted on a smaller scale may also potentially yield models with a higher accuracy at a higher resolution.

## 5. Conclusions

The present work represents the first study that used the application of GIS to predict the distribution of bacteria in the future and it provides valuable insights into the potential impact of climate change on the distribution of *A. baumannii*, a bacterial species that poses a significant threat to human health. The study applies MaxEnt modeling techniques to predict the current and future distribution of *A. baumannii* under four different climate scenarios. The generated risk maps suggest that *A. baumannii* is likely to expand its range in the future, especially through Europe, which could have serious implications for public health. The study highlights the need for further research and action to mitigate the impact of climate change on the distribution of pathogenic microorganisms.

## Figures and Tables

**Table 1:** Contribution and permutation importance of the most important contributors to the global distribution of *A. baumannii*.

Variable	Percent Contribution	Permutation Importance
Annual mean temperature (bio_1)	9.5%	26.1%
Minimum temperature of the coldest month (bio_6)	12.6%	26.1%
Mean temperature of the coldest quarter (bio_11)	37.8%	32.9%
Annual precipitation (bio_12)	40.2%	14.9%

## Data Availability

All data are available throughout the manuscript.

## Supplementary Materials

The following supporting information can be downloaded at: https://www.mdpi.com/article/10.3390/microorganisms11092174/s1, Table S1: The 19 bioclimatic variables used to generate the prediction distribution maps of *A. baumannii*; Figure S1: Environmental envelope model of recorded points of *A. baumannii*, the envelope showing the wide range of annual precipitation (bio_12) and annual mean temperature (bio_1).
