# Supplementary material for: Global Risk Maps of Climate Change Impacts on the Distribution of Acinetobacter baumannii Using GIS
Source: Microorganisms. 2023 Aug 28;11(9):2174. doi: 10.3390/microorganisms11092174 (PMC10535618; doi:10.3390/microorganisms11092174)
Supplement: Supplementary file 1 [file microorganisms-11-02174-s001.zip › Figure S1.pdf]

Figure S1: Environmental envelope model of recorded points of *A. baumannii*, the envelope showing the wide range of annual precipitation (bio_12) and annual mean temperature (bio_1).

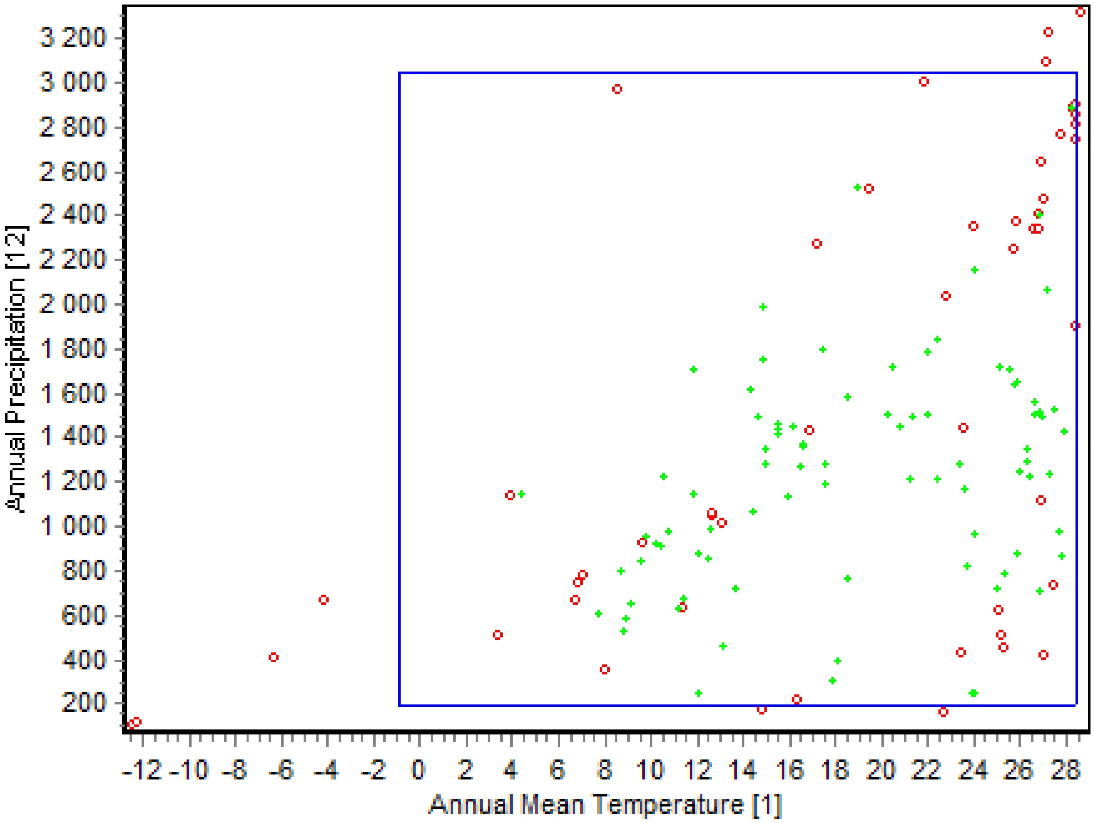

* The test done using DIVA-GIS V7.1
