# Supplementary material for: Global Risk Maps of Climate Change Impacts on the Distribution of Acinetobacter baumannii Using GIS
Source: Microorganisms. 2023 Aug 28;11(9):2174. doi: 10.3390/microorganisms11092174 (PMC10535618; doi:10.3390/microorganisms11092174)
Supplement: Supplementary file 1 [file microorganisms-11-02174-s001.zip › Table S1.pdf]

Table S1. The 19 bioclimatic variables used to generate the prediction distribution maps of *A. baumannii*

variable	Description
Bio 1	Annual mean temperature
Bio 2	Mean diurnal range
Bio 3	Isothermality (bio2\bio 7) (*100)
Bio 4	Temperature seasonality (standard deviation*100)
Bio 5	Maximum temperature of the warmest month
Bio 6	Minimum temperature of the coldest month
Bio 7	Temperature annual range
Bio 8	The mean temperature of the wettest quarter
Bio 9	The mean temperature of the driest quarter
Bio 10	The mean temperature of the warmest quarter
Bio 11	The mean temperature of the coldest quarter
Bio 12	Annual precipitation
Bio 13	Precipitation of the wettest month
Bio 14	Precipitation of the driest month
Bio 15	Precipitation seasonality
Bio 16	Precipitation of the wettest quarter
Bio 17	Precipitation of the driest quarter
Bio 18	Precipitation of the warmest quarter
Bio 19	Precipitation of the coldest quarter
